# Experience Matters: An Application of the Operative Value Index for Lumbar Fusions

**DOI:** 10.1007/s10143-025-03730-8

**Published:** 2025-07-22

**Authors:** Danyal Quraishi, Advith Sarikonda, D. Mitchell Self, Steven Glener, Arbaz Momin, Emily L. Isch, Ashmal Sami Kabani, Matthews Lan, Nicholas Clark, Ashwini Sharan, Srinivas Prasad, Jack Jallo, Joshua Heller, Alexander R. Vaccaro, James Harrop, Ahilan Sivaganesan

**Affiliations:** 1 Department of Surgery, Drexel University College of Medicine, Philadelphia, PA USA; 2 Department of Neurosurgery, Thomas Jefferson University and Jefferson Hospital for Neuroscience, Philadelphia, PA USA; 3 Department of General Surgery, Thomas Jefferson University Hospital, Philadelphia, PA USA; 4 Department of Spine Surgery, Hospital for Special Surgery at Naples Comprehensive Health, Naples, FL USA; 5 Rothman Orthopedic Institute, Philadelphia, PA USA

**Keywords:** Transforaminal lumbar interbody fusion (TLIF), Intraoperative costs, Operative times, Time-Driven Activity-Based costing (TDABC), Spinal fusion surgery, Healthcare costs, Surgeon experience, Case volume, Health economics, Bundled payments, Operating room efficiency

## Abstract

**Introduction:**

As healthcare systems transition to value-based care models, it is critical to understand factors influencing costs and outcomes in neurosurgery. This study utilizes Time-Driven Activity-Based Costing (TDABC) to assess the impact of surgeon experience and case volume on costs, clinical outcomes, and operative value in lumbar fusions. While TDABC has been applied in various surgical specialties, its use in neurosurgery, particularly in analyzing surgeon-specific factors, remains limited.

**Methods:**

A retrospective cohort study was conducted at a single center, analyzing lumbar instrumented arthrodesis procedures from 2017 to 2019. Primary variables included surgeon experience (years) and case volume (annual procedures). Confounding variables such as patient demographics, comorbidities, surgery type, and levels fused were considered. Intraoperative cost data were sourced from electronic medical records and departmental sources. Clinical outcomes were measured using the Oswestry Disability Index (ODI), and operative value was defined by the Operative Value Index (OVI), defined as the percent improvement in ODI per $1000 spent intraoperatively. Analysis of covariance (ANCOVA) examined the relationships between surgical costs, surgeon experience, case volume, and clinical outcomes.

**Results:**

Among 291 surgeries, surgeons with ≥ 15 years of experience had lower mean surgery costs ($16,071.78 vs. $22,259.71, *p* < 0.001) and higher OVI scores (1.81 vs. 1.2, *p* = 0.033) compared to less experienced surgeons. High-volume surgeons (≥ 100 annual cases) showed greater ODI improvements (34.90 vs. 22.07, *p* = 0.022) and higher OVI scores (2.22 vs. 1.01, *p* = 0.016) compared to lower-volume surgeons. Procedure type and levels fused significantly influenced surgery costs (*p* < 0.001) and OVI (*p* < 0.001).

**Conclusions:**

This study is among the first to apply TDABC in evaluating the impact of surgeon experience and case volume on costs and outcomes in lumbar fusion procedures. Findings suggest that surgeons with ≥ 15 years of experience and high case volumes (≥ 100 annual cases) enhance operative value by reducing costs and improving ODI, respectively. Encouraging specialization and maintaining high case volumes may enhance cost-effectiveness and patient outcomes in healthcare systems.

## Introduction

With ever-increasing expenses and dwindling viable resources, healthcare systems worldwide are becoming increasingly focused on providing high value care [1]. It is well established that of the many moving parts in a hospital system, the operating room (OR) is a resource-dense environment that is consistently cited as one of the most expensive areas in a patient’s care [2]. Amongst the surgical fields, neurosurgery is notorious for complex procedures requiring high-cost technology [3–5]. To better illuminate the drivers of cost in these procedures, variations between a neurosurgeon’s experience and case volume has been investigated in prior literature [6]. Recent studies have found that higher neurosurgeon case volume and greater years of experience are associated with significantly lower risks of postoperative complications, shorter length of stay, reduced hospital costs, and lower rate of readmission and reoperation [6–9]. 

However, hospital cost in most studies is often a function of cost-to-charge ratios and/or relative value units, which are not accurate representations of the true expenses associated with care delivery. TDABC has emerged as an innovative micro-costing tool that enables healthcare providers to obtain precise data on the costs of care delivery across the entire patient journey, and has only recently been applied in the field of neurosurgery [6, 10–14]. Unlike traditional costing methods, TDABC provides a detailed analysis of resource utilization, personnel involvement, and time spent on each activity within the care process. By assigning a cost to each minute of resource use, TDABC enables healthcare systems to identify cost-saving opportunities, improve process efficiency, and ultimately contribute to higher value in patient care. The use of TDABC is especially important considering health care systems’ emphasis on Value-Based Health Care (VBHC), in which the primary goal is to maximize patient outcomes relative to incurred cost [1]. 

Although certainly beneficial, cost transparency alone is insufficient in implementing high value care to neurosurgical patients. A patient’s postoperative clinical outcome must also be considered. To better grasp both factors, we integrate TDABC with procedure-specific Patient Reported Outcomes (PROs). In this study, we utilize the Oswestry Disability Index (ODI) as our specified PRO. Specifically, we employ a novel Operative Value Index (OVI), defined as a percent improvement in ODI per $1000 spent intraoperatively. This novel metric has been recently incorporated into neurosurgical studies to better illustrate high-value findings [15–19]. Through this work, we hope to assess whether increased surgeon experience and case volume truly provides improved outcomes per dollar spent.

### Primary objective

The study aims to analyze how surgeon experience (measured in years) and case volume (measured in number of procedures performed annually) affect the cost-effectiveness of neurosurgical interventions. By implementing TDABC in the evaluation process, the study seeks to accurately measure the costs associated with various neurosurgical procedures and understand how these costs are influenced by the surgeon’s experience level and procedural volume. Combining this with an evaluation of the PROs (i.e. ODI), we are better able to properly assess whether increased surgeon experience and case volume truly provides improved outcomes per dollar spent.

## Methods

### Patient population

A retrospective review of the electronic medical record (EMR) was conducted for all patients who underwent lumbar instrumented arthrodesis procedures from 2017 to 2019 at our institutional enterprise.

### General data collection

This study was approved by the Thomas Jefferson University institutional review board under a universal Quality Improvement protocol (#21D.1240). Data collection included baseline patient demographic information, such as age, race, sex, comorbidities, and smoking status. Additionally, procedural data was collected, including: the surgeon performing procedure, the type of surgery performed, number of levels fused, staff utilized, and time associated with each procedure. Intraoperative cost data were sourced from electronic medical records and departmental sources. Clinical outcomes were measured using ODI, and operative value was defined by OVI, representing the percent improvement in ODI per $1000 spent intraoperatively.

### Surgeon characteristics and classification

**Fig. 1:**
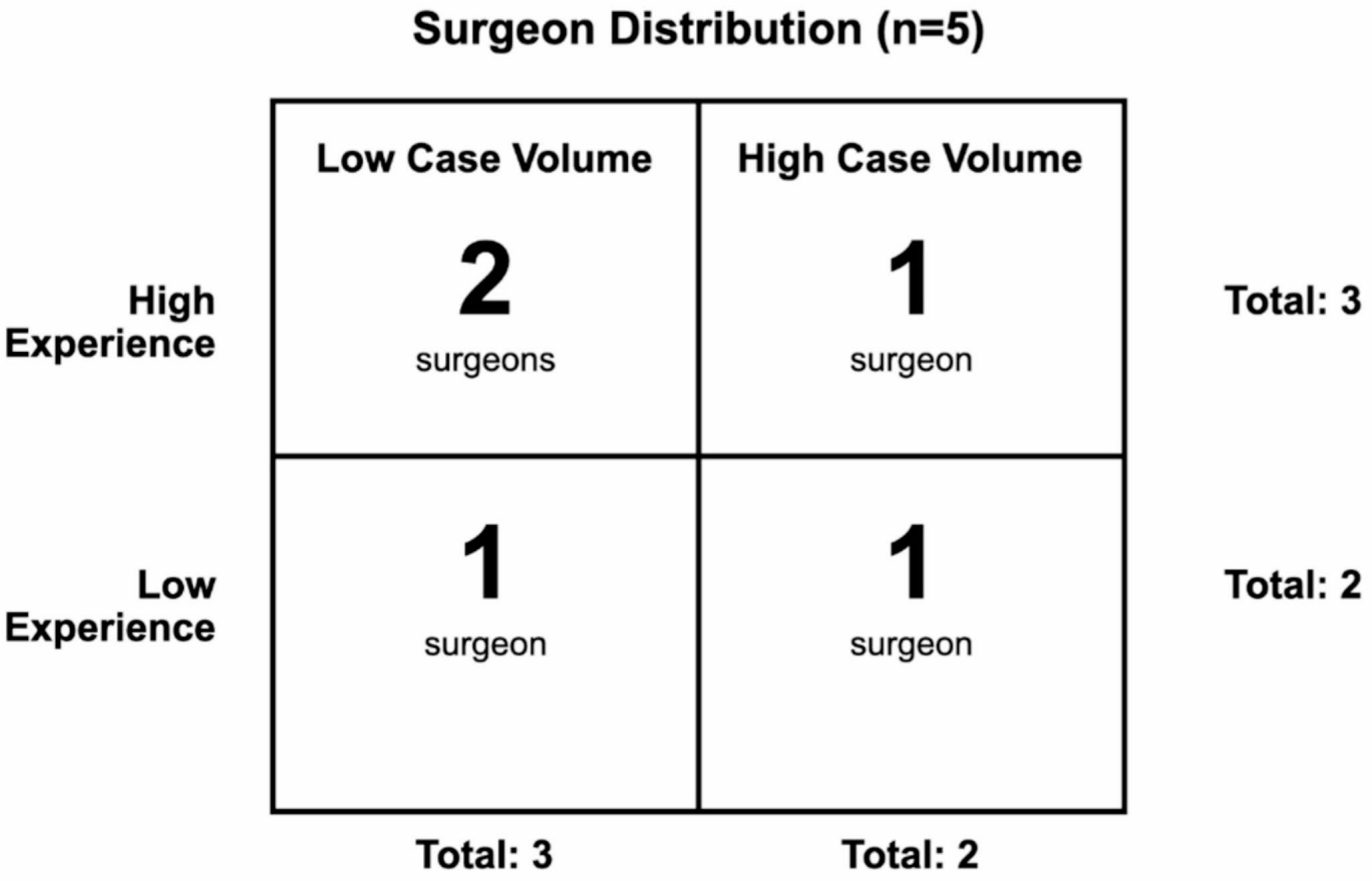
Distribution of surgeons by experience level and case volume

Case volume refers to the surgeon’s average annual lumbar fusion cases sourced from institutional data that spans from their fellowship end date to 2019. 4 neurosurgeons are fellowship trained in the spine, while one is fellowship trained in neurotrauma and critical care. Of the 5 surgeons, 2 were categorized as low experience and 3 were categorized as high experience. 3 surgeons were categorized as low case volume surgeons and 2 were categorized as high case volume surgeons. Figure 1 displays the distribution amongst experience and case volume across the 5 surgeons.

### Time-driven activity-based costing methodology

**Fig. 2:**
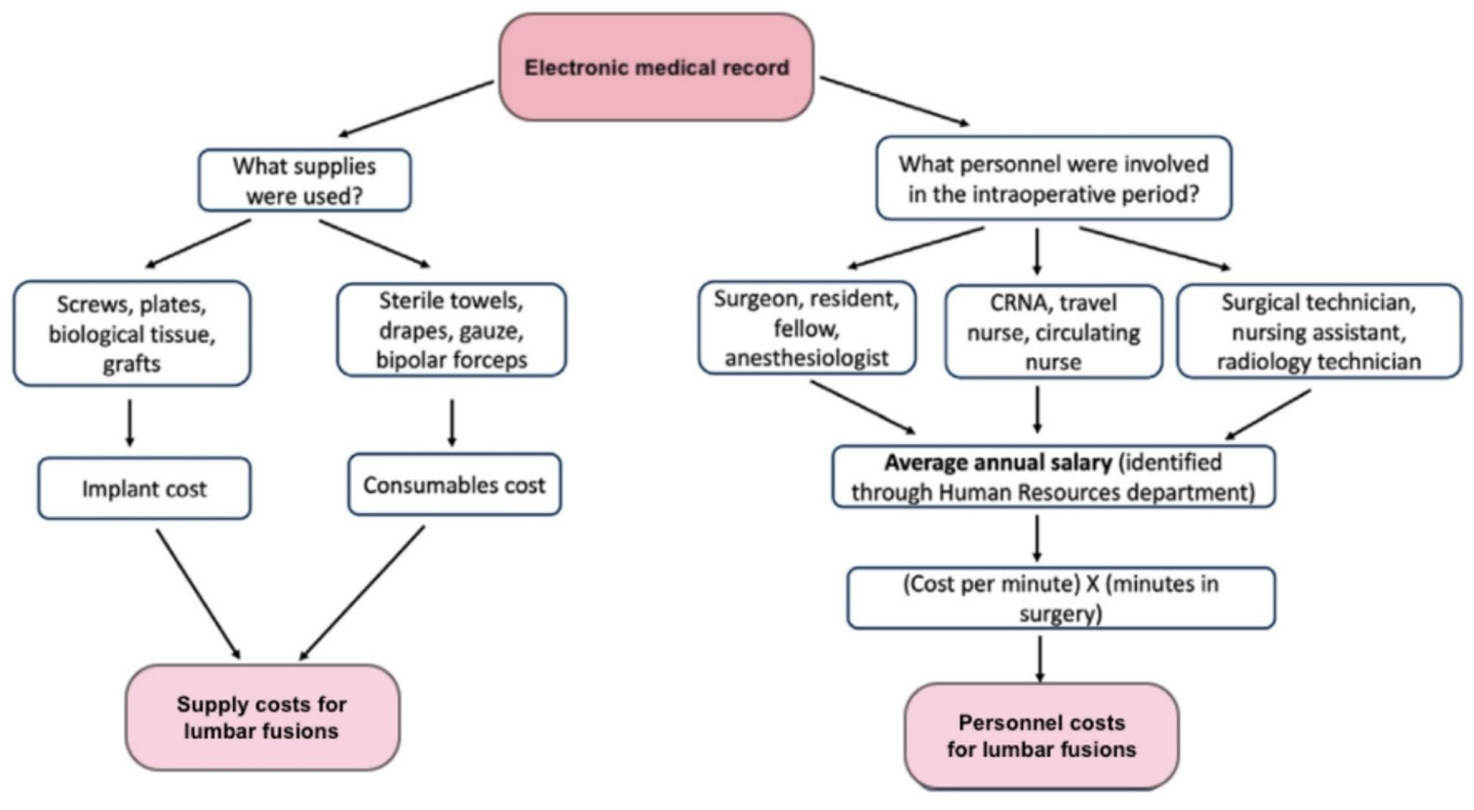
Time driven activity based costing methodology to calculate supply and personnel cost, the 2 major drivers of intraoperative cost

Cost data was collected using the TDABC methodology. The 2 major components of the total cost using this methodology are supply cost and personnel cost. To determine personnel cost, we first utilized the EMR to identify the exact personnel types documented in each surgical episode. Then, we determined the prototypical annual salary for each of these personnel types. Using these annual salaries, we then calculated the cost per minute per personnel type, which was then multiplied by the minutes spent in the OR. This constituted total personnel cost for each case. To calculate supply cost, we queried the EMR to identify both implant-related surgical supplies (such as screws, plates, biologic tissue, and grafts) as well as consumables (such as sterile towels, drapes, and bipolar forceps) which were used intraoperatively. The cost associated with use of each of these resources was determined through consultation with our institution’s business operations department. Supply cost were therefore an aggregate of implant related costs and consumables costs. A visual summary of the methodology used to calculate supply and personnel cost, the two primary drivers of total intraoperative cost, is shown in Fig. 2. Through consultation with departments such as pharmacy and plant operations, we also extracted medication cost, sterilization cost, overhead cost, and turnover cost (defined as the cost associated with replacement of materials from one operation to the next for each procedure from the EMR). The total cost of surgery was therefore a composite of supply cost, personnel cost, medication cost, turnover cost, sterilization cost. and overhead cost.

### Statistical analysis

We used the RStudio software (Version 2024.04.2 + 764) to conduct statistical analysis. Cases were divided according to the surgeon’s experience and case volume, with two subgroups for each variable. The distribution of experience was divided into a low experience group (less than 15 years of experience) and a high experience group (greater than or equal to 15 years of experience). The distribution of case volume was divided into a low case volume group (less than 100 cases) and a high case volume group (greater than or equal to 100 cases). The threshold of 100 cases represented a natural division in our data that created meaningful and balanced cohorts for comparison. When examining the distribution of case volumes among our surgeons, we observed a bimodal pattern with a clear separation around this threshold. This threshold also provided sufficient power for statistical analysis while maintaining clinically meaningful distinctions between groups. Analysis of covariance (ANCOVA) was used to evaluate the independent effects of both surgeon experience and case volume on total surgical costs, ODI, and OVI while controlling for confounding variables such as patient demographics, comorbidities, surgery type, and number of levels fused.

## Results

### Descriptive statistics

**Fig. 3:**
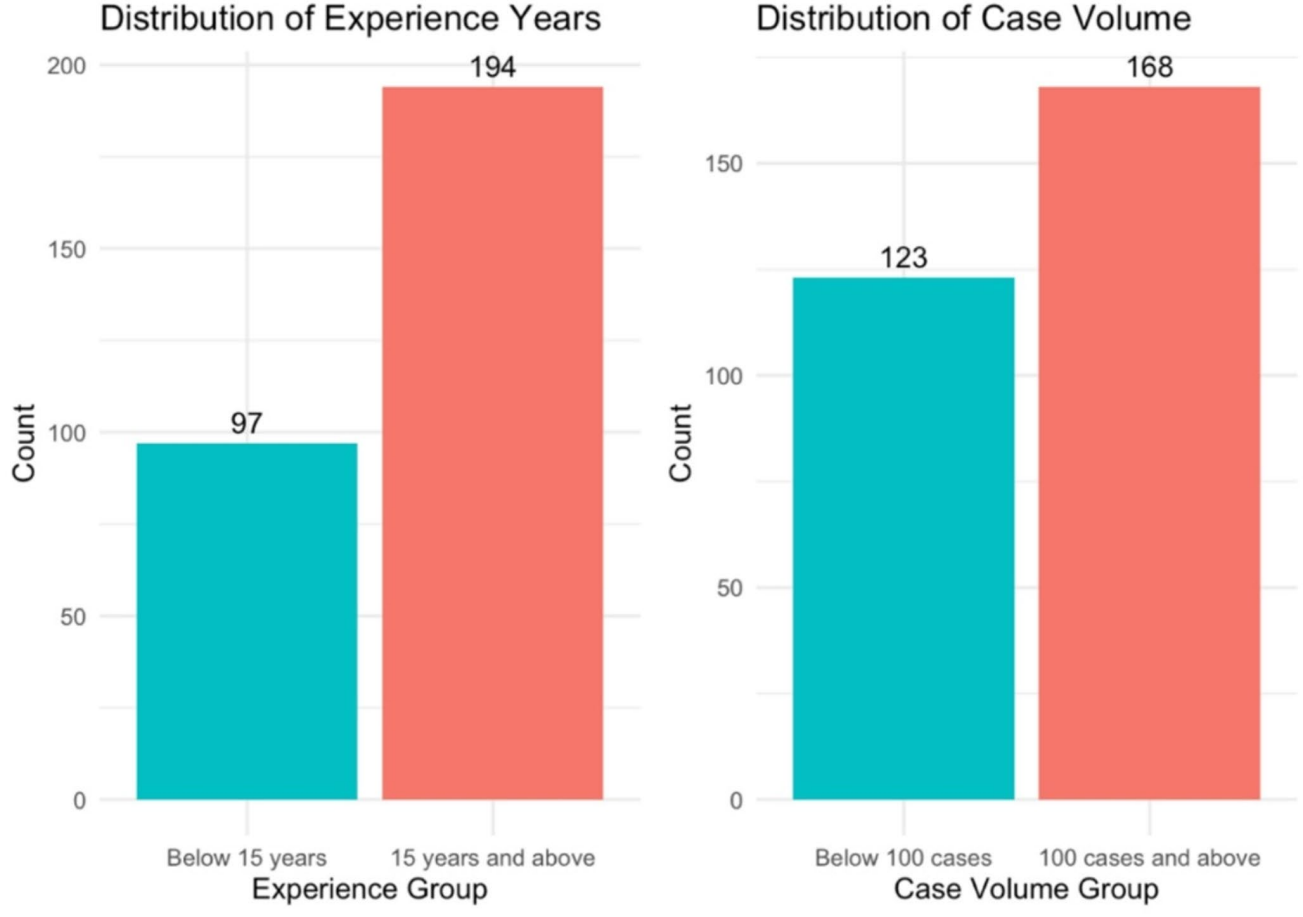
Count distribution of surgeon experience and case volume

A total of 291 patients underwent lumbar interbody fusions between 2017 and 2019. These procedures were performed by 5 surgeons. The study’s mean number of levels fused, BMI, and age was 2.08 ± 1.65, 30.25 ± 5.85, and 62.69 ± 11.89, respectively. 8 procedures were done using minimally invasive techniques, and 11 cases involved use of robotics. All other cases were employed using an open technique across all procedures. Further characteristics including comorbidities, race, and procedure characteristics are shown in Table 1. There were 194 high experience cases and 97 low experience cases. There were 168 high volume cases and 123 low volume cases. The distribution of experience and case volume are shown in Fig. 3.

Our analysis revealed several significant differences between surgeon groups. For smoking status, high-experience surgeons had a significantly higher proportion of smokers in their patient cohort compared to low-experience surgeons (18.6% vs. 8.2%, *p* = 0.03). Regarding comorbidities, high-volume surgeons treated a significantly lower proportion of patients with diabetes mellitus compared to low-volume surgeons (25% vs. 38.2%, *p* = 0.02). There were also significant racial differences in patient distribution, with high-volume surgeons treating a higher proportion of White/Caucasian patients (92.3% vs. 82.9%, *p* = 0.02) and a lower proportion of Black/African American patients (6.0% vs. 14.6%, *p* = 0.02) compared to low volume surgeons.

Procedure selection also differed significantly between groups. High-experience surgeons performed a significantly higher percentage of transforaminal lumbar interbody fusion (TLIF) procedures compared to low-experience surgeons (69% vs. 36.1%, *p* < 0.001), while low-experience surgeons performed significantly more lateral lumbar interbody fusion (XLIF) (20.6% vs. 1.5%, *p* < 0.001) and anterior lumbar interbody fusion (ALIF) (17.5% vs. 2.6%, *p* < 0.001) procedures. Similarly, high-volume surgeons performed more TLIF procedures (67.9% vs. 44.7%, *p* < 0.001) and fewer XLIF procedures (4.2% vs. 13%, *p* = 0.01) compared to low-volume surgeons.

**Table 1:** Demographic characteristics of patients

Characteristic	High Experience (≥ 15 years)	Low Experience (< 15 years)	*p*-value	High Volume (≥ 100 cases)	Low Volume (< 100 cases)	*p*-value
Patient Demographics						
**n**	194	97	-	168	123	-
Age (mean ± SD)	69.2 ± 11.8	68.9 ± 12.3	0.84	69.7 ± 12.6	68.3 ± 11.0	0.32
BMI (mean ± SD)	30.1 ± 5.3	30.6 ± 6.8	0.49	30.1 ± 6.2	30.5 ± 5.3	0.55
Male, n (%)	86 (44.3)	40 (41.2)	0.12	66 (39.3)	60 (48.8)	0.73
Female, n (%)	108 (55.7)	57 (58.8)	0.12	102 (60.7)	63 (51.2)	0.73
Smoker, n (%)	36 (18.6)	8 (8.2)	**0.03**	22 (13.1)	22 (17.9)	0.33
**Comorbidities**						
Diabetes mellitus, n (%)	60 (31.0)	29 (30.0)	0.96	42 (25)	47 (38.2)	**0.02**
Osteoporosis, n (%)	70 (36.1)	42 (43.3)	0.29	64 (38.1)	48 (39.0)	0.97
**Race**						
White or Caucasian, n (%)	170 (87.6)	87 (89.7)	0.75	155 (92.3)	102 (82.9)	**0.02**
Black or African American, n (%)	19 (9.8)	9 (9.3)	1.0	10 (6.0)	18 (14.6)	**0.02**
Hispanic, n (%)	1 (0.5)	0 (0)	-	1 (0.6)	0 (0)	-
Asian, n (%)	2 (1)	1 (1)	-	1 (0.6)	2 (1.6)	-
Other, n (%)	1 (0.5)	0 (0)	-	0 (0)	1 (0.8)	-
**Procedure Characteristics**						
Number of levels fused (mean ± SD)	2.06 ± 1.68	2.12 ± 1.58	0.76	2.0 ± 1.6	2.2 ± 1.7	0.31
**Procedure Type**						
TLIF, n (%)	134 (69)	35 (36.1)	**< 0.001**	114 (67.9)	55 (44.7)	**< 0.001**
Anterior-Posterior, n (%)	25 (12.9)	7 (7.2)	0.21	18 (10.7)	14 (11.4)	1.0
PLIF, n (%)	21 (10.8)	12 (12.4)	0.85	16 (9.5)	17 (13.8)	0.34
XLIF, n (%)	3 (1.5)	20 (20.6)	**< 0.001**	7 (4.2)	16 (13)	**0.01**
ALIF, n (%)	5 (2.6)	17 (17.5)	**< 0.001**	10 (6.0)	12 (9.8)	0.32
Not Reported, n (%)	5 (2.6)	5 (5.2)	0.43	3 (1.8)	7 (5.7)	0.14

**Fig. 4:**
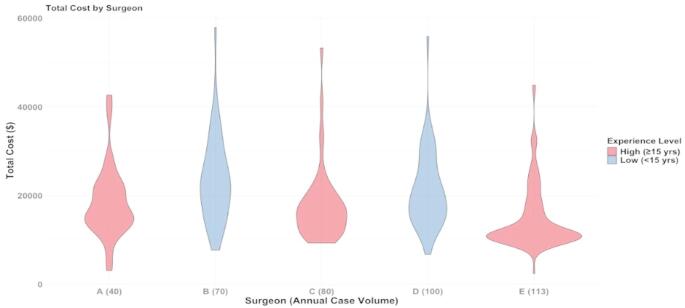
Total cost distribution across surgeons

**Fig. 5:**
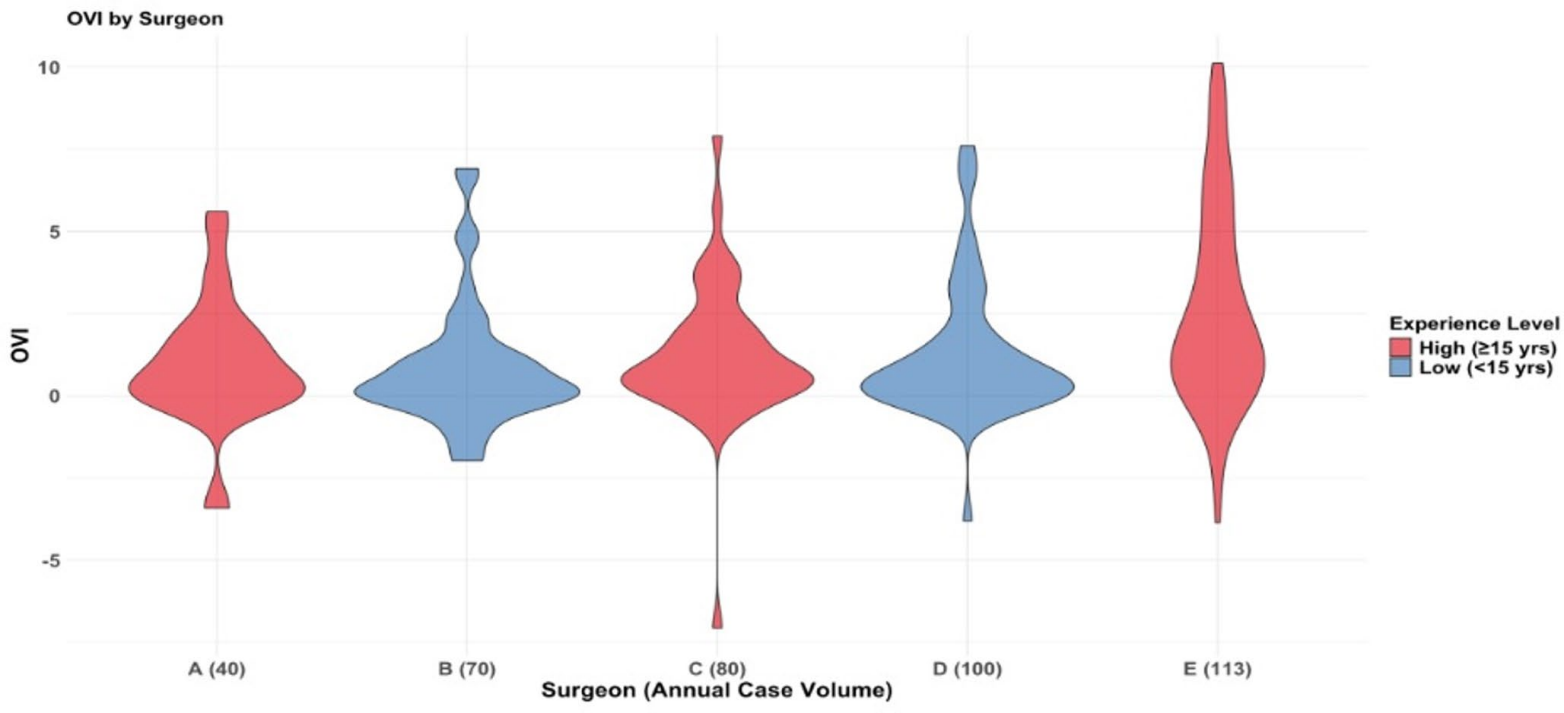
OVI distribution across surgeons

The overall mean total cost for all TLIF surgeries was $18,134.42 +/- $8,709.37. The mean total cost of surgery for each cohort was the following: $16,071.78 +/- $8,340.33 for high surgeon experience; $22,259.71 +/- $8,793.87 for low surgeon experience; $16,707.11 +/- $8,129.95 for high case volume, $14,696.16 +/- $9,121.05 for low case volume. More information about the mean cost and operative time associated with each group is shown in Tables 2 and 3. Violin plots were created to visualize the distribution of total cost and OVI for each surgeon, which are seen in Figs. 4 and 5 respectively.

**Table 2:** Descriptive statistics for cost and time for surgeon experience groups

Cohort	*n* (%)	Total supply Cost per Case	Total Personnel Cost per Case	Total Cost of Surgery	Minutes in Surgery	Total minutes in the Operating Room	Total minutes in the Operating Room not Operating
High Surgeon Experience (> 15 years)	194 (66.7%)	$11,550.74	$ 4,023.80	$16,071.78	242.7	324.4	81.7
Low Surgeon Experience (< 15 years)	97 (33.3%)	$16,495.10	$ 4,986.90	$22,259.71	298.9	398.9	100.0
Total	291 (100%)	$13,212.04	$4,344.84	$18,134.42	261.4	349.2	87.8

**Table 3:** Descriptive statistics for cost and time for case volume groups

Cohort	*n* (%)	Total supply Cost per Case	Total Personnel Cost per Case	Total Cost of Surgery	Minutes in Surgery	Total minutes in the Operating Room	Total minutes in the Operating Room not Operating
High Case Volume (> 100 cases)	168 (57.7%)	$12,119.57	$3,852.74	$16,707.11	226.2	307.0	80.8
Low Case Volume (< 100 cases)	123 (42.3%)	$14,696.16	$5,016.98	$20,083.92	309.5	406.9	97.4
Total	291 (100%)	$13,212.04	$4,344.84	$18,134.42	261.4	349.2	87.8

**Fig. 6:**
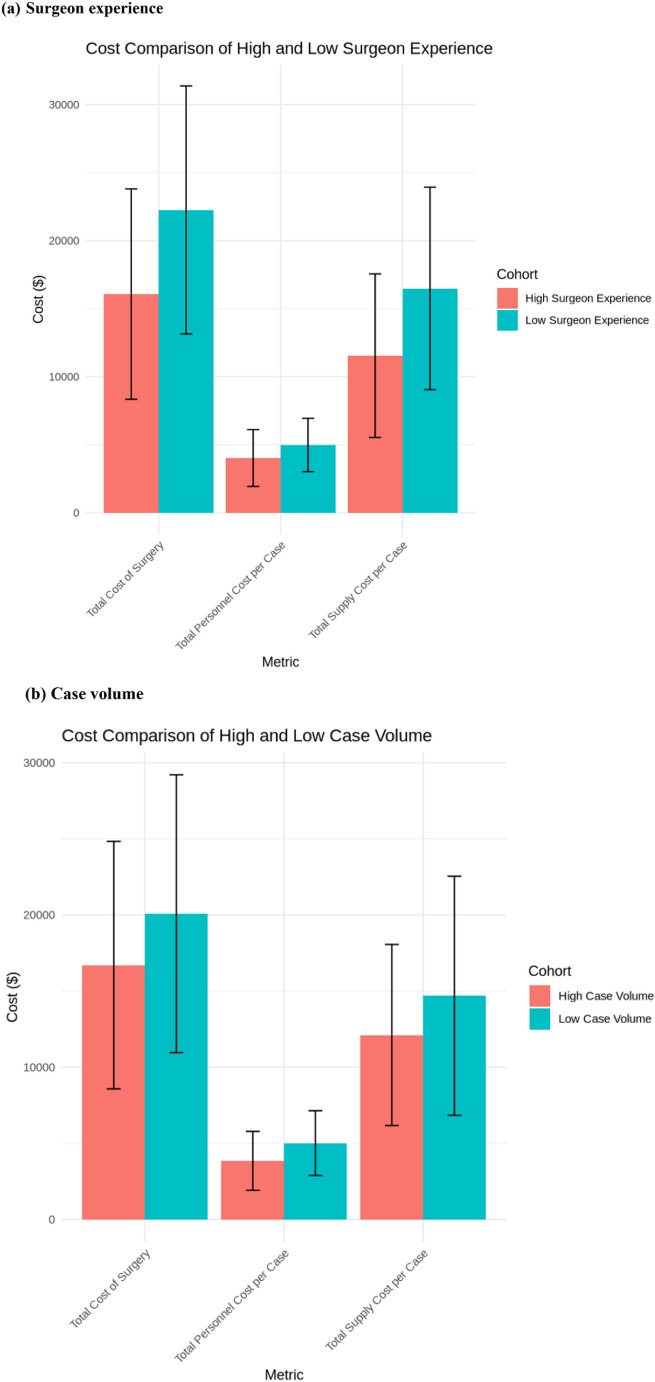
Comparisons of the major drivers of cost among the surgeon experience and case volume groups

Additionally, the significant contributors to cost, consisting of supplies and personnel cost, among the cohorts is shown in Fig. 6.

### Analysis of covariance (ANCOVA)

#### Cost

The ANCOVA analyses revealed that surgeon experience significantly influenced surgery costs, with surgeons having 15 or more years of experience demonstrating significantly lower mean costs compared to less experienced surgeons (F = 19.08, *p* < 0.001). Case volume did not significantly influence total surgery cost. (F = 1.69, *p* = 0.19). Additionally, procedure type and the number of levels fused were significant factors influencing surgery costs (F = 9.93, *p* < 0.001) and OVI (F = 7.27, *p* < 0.001), respectively.

#### Change in ODI

High-case volume surgeons (≥ 100 cases) showed significantly greater ODI improvements compared to lower-volume surgeons (F = 5.30, *p* = 0.022). Surgeon experience did not significantly influence change in ODI (F = 1.85, *p* = 0.175).

#### OVI

**Fig. 7:**
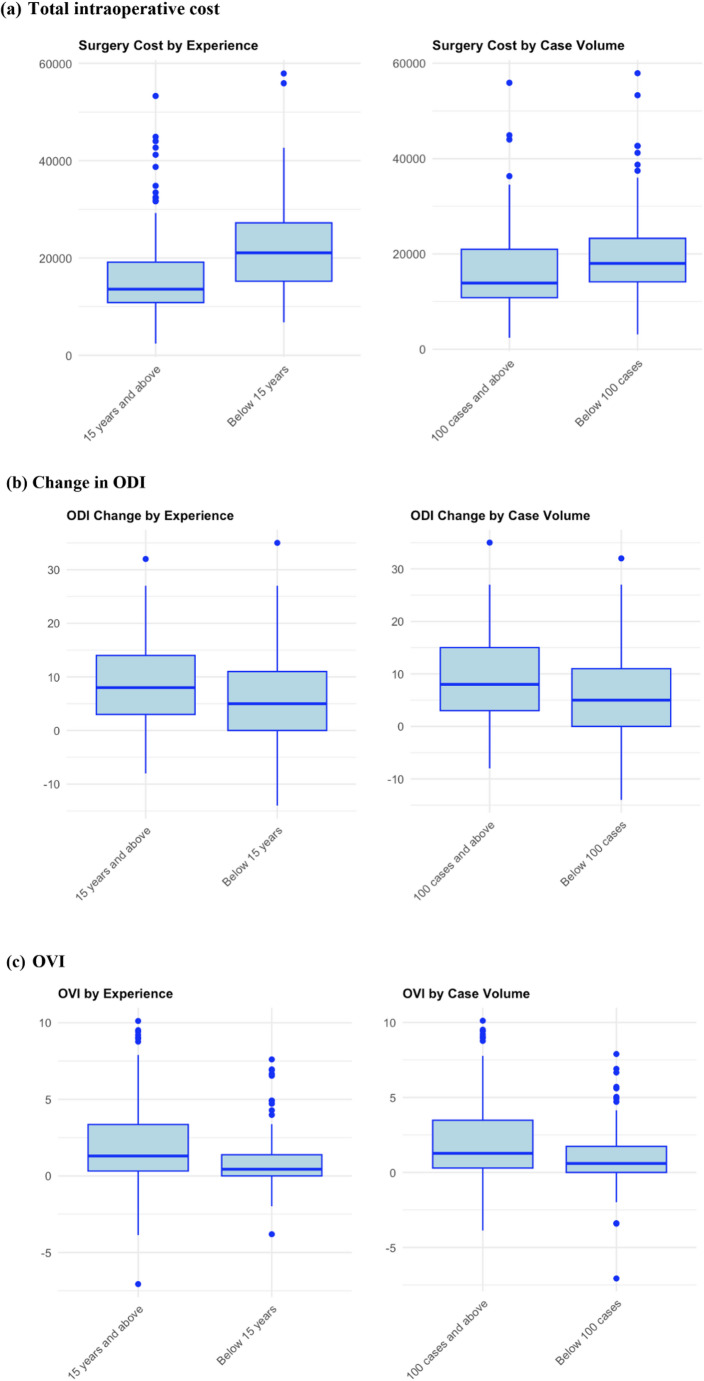
Surgeon experience and case volume on total cost, change in ODI, and OVI

Both surgeon experience and case volume significantly affected the Operative Value Index (OVI), with more experienced and high-volume surgeons achieving higher OVI scores (Experience: F = 4.57, *p* = 0.033; Volume: F = 5.86, *p* = 0.016). Visualizations are provided in Fig. 7.

## Discussion

### Interpretation of findings

The results of this study provide significant insight into the impact of surgeon experience and case volume on both the costs and outcomes of lumbar fusion surgeries, utilizing Time-Driven Activity-Based Costing (TDABC) as a micro-costing tool. The findings support and extend existing literature, particularly within the broader fields of orthopedic and cardiac surgery, but they offer new perspectives specific to neurosurgery.

### Surgeon experience and total costs

Surgeon experience was found to be a critical factor in reducing total surgical costs. Surgeons with more than 15 years of experience had significantly lower costs per procedure compared to their less experienced counterparts. This is consistent with studies in other surgical disciplines, where experienced surgeons are associated with shorter operative times, reduced complications, and more efficient resource use [11, 15]. Experienced surgeons tend to be more proficient in decision-making, possibly due to their familiarity with complex procedures, which minimizes unnecessary use of operating room (OR) time and resources [4]. As demonstrated in this study, the costs for surgeons with more than 15 years of experience averaged $16,071.78 per case, compared to $22,259.71 for less experienced surgeons. This reduction in costs underscores the importance of surgeon experience in promoting cost-effective surgical care.

### Case volume and clinical outcomes

The findings also highlight the strong correlation between high case volumes and improved clinical outcomes. Surgeons performing more than 100 procedures showed greater improvements in patient-reported outcomes such as the ODI. This aligns with the literature indicating that high-volume surgeons are generally associated with better clinical outcomes, possibly due to increased technical proficiency and process standardization [9]. High case volume may enhance both teamwork within the surgical team and perioperative care management, contributing to higher-quality outcomes for patients [11]. This study reinforces this correlation, showing that high-volume surgeons achieve higher OVI scores, reflecting better outcomes per dollar spent.

### Surgeon preferences and patient selection patterns

The significant differences in patient characteristics and procedure selection between surgeon groups may have influenced our findings. The higher proportion of patients with diabetes in the low-volume surgeon group could have contributed to increased complexity and potentially higher costs. Similarly, the significant differences in racial distribution between high and low-volume surgeons highlight potential selection biases that may affect outcomes.

The substantial differences in procedure selection between experienced and less experienced surgeons, as well as between high and low-volume surgeons, are particularly noteworthy. Experienced and high-volume surgeons demonstrated a clear preference for TLIF procedures, while less experienced surgeons more frequently performed XLIF and ALIF procedures. This difference in approach may reflect training backgrounds, comfort levels with different techniques, or strategic responses to their respective patient populations. The greater use of TLIF procedures by high-experience and high-volume surgeons may be associated with standardization of their surgical approach, potentially contributing to the improved efficiency and reduced costs observed in these groups.

While our ANCOVA analyses controlled for procedure type, the strong preference patterns observed suggest that surgeon experience and volume may shape decision-making in ways that extend beyond the variables we measured. The interaction between surgeon characteristics, procedure selection, and patient outcomes warrants further investigation.

### Alignment with existing literature

The study’s results support findings previously demonstrated in the fields of both orthopedic and cardiac surgery, where TDABC has been effectively employed to reduce costs and enhance outcomes. Specifically, the correlation between surgeon experience and lower costs has been a consistent finding across surgical specialties [4]. However, this study contributes novel insights into how these factors influence outcomes specifically in the realm of neurosurgical procedures like lumbar fusions, where time-intensive technology and resource use make experience a key driver of efficiency [10]. 

Moreover, the impact of high case volume on clinical outcomes, such as improvement in ODI, reinforces the evidence that repetition and procedural familiarity contribute significantly to enhanced care delivery [9]. This study diverges from some existing literature by emphasizing that in neurosurgery, where technology-driven and time-dependent costs dominate, the experience of the surgeon has an outsized impact on overall cost savings. Unlike other surgical fields, consumables are not the primary cost driver in these procedures [11]. 

## Implications for practice

### Training and mentorship

The study’s findings suggest that healthcare systems should place a greater emphasis on the training and mentorship of less experienced surgeons. By fostering mentorship programs where experienced surgeons guide junior colleagues, institutions can expedite the learning curve and help younger surgeons minimize costs without compromising care quality [10]. Such mentorship programs could be particularly beneficial in spine surgery, where both operative time and complication rates are highly sensitive to surgeon experience.

### Value-Based healthcare (VBHC) and TDABC

The application of TDABC in this study underscores its utility in VBHC models. TDABC enables precise identification of cost drivers and allows for more granular cost management, aligning with VBHC’s goal of maximizing outcomes relative to cost [4]. As healthcare systems continue to shift towards value-based payment models, the insights provided by TDABC are paramount in optimizing both care quality and financial performance. In particular, experienced surgeons who consistently deliver cost-effective care could be viewed as valuable assets under bundled payment models [11]. 

### Standardization of care

This study also highlights the potential for developing standardized care pathways based on cost and outcomes data. Surgeons and institutions could adopt these pathways to ensure consistent delivery of high-value care, reducing variability in both costs and outcomes [15]. By leveraging the data from TDABC, healthcare providers can refine these protocols to streamline surgical processes, enhance efficiency, and improve patient outcomes.

### Limitations

This study, while offering valuable insights, has limitations that must be acknowledged. First, the retrospective nature of the data introduces the potential for biases in time estimation and resource use [11]. Additionally, a high case volume threshold may vary across institutions. This binary split, while necessary for our analysis, simplifies what is likely a continuous relationship between volume and outcomes. The relatively small cohort size of 291 procedures performed by 5 surgeons may affect the generalizability of our findings. Furthermore, the study was conducted at a single institution, which may limit the generalizability of the findings to other healthcare settings. Future research should aim to validate these results across multiple institutions and within diverse healthcare systems to improve the applicability of the findings.

Our analysis revealed significant differences in baseline patient characteristics and procedure selection between surgeon groups, introducing potential confounding factors despite our statistical controls. The significantly higher proportion of diabetic patients among low-volume surgeons and differences in racial distribution between volume groups may reflect underlying referral patterns or case selection biases that could influence outcomes independently of surgeon volume or experience. Similarly, the marked differences in procedure selection between groups may reflect training backgrounds or evolving practice patterns rather than direct effects of experience or volume.

While our ANCOVA model attempted to control for these factors, the small sample size limits our ability to fully account for all potential confounders simultaneously. Additionally, the institutional nature of practice patterns may limit generalizability to other settings with different patient populations or procedural preferences. Future larger, multi-institutional studies with more diverse surgeon cohorts would help validate whether the observed relationships persist when controlling for these variables more comprehensively.

Another limitation of our study is the lack of detailed data on bone morphogenetic protein (BMP) utilization. BMP represents a substantial cost factor in spinal fusion procedures and could potentially explain some of the observed cost differences between surgeon experience groups. Our dataset did not specifically capture BMP usage, preventing us from analyzing its specific contribution to the overall cost differences. Future studies should include granular documentation of BMP2 to better elucidate their impact on the cost variation between more and less experienced surgeons.

While this study highlights the cost-effectiveness of experienced, high-volume surgeons using TDABC, it is essential to consider broader TDABC applications and limitations in achieving truly patient-centered, value-based health care. TDABC’s primary strength lies in its ability to pinpoint resource costs at a granular level, yet it may benefit from integrating more patient-centered metrics, such as readmission rates and emergency department (ED) visits, which directly reflect post-operative patient outcomes and satisfaction [20]. As Kaplan and Porter (2011) have emphasized, true value-based care requires not only cost reduction but also the improvement of outcomes that matter to patients, a goal that could be advanced by aligning TDABC with metrics that capture broader patient experiences [21]. These additions could enhance the alignment of TDABC with VBHC goals, ensuring cost measurements that resonate with both clinical efficiency and patient experience.

Furthermore, the resource-intensive nature of TDABC, particularly in data collection and time estimation, presents practical challenges. As Keel et al. (2017) suggest, future implementations may benefit from process mining or automated data collection methods within electronic medical records, which can streamline these processes [20]. This could be particularly advantageous in neurosurgery, where high personnel costs drive overall expenses. Additionally, variations in indirect cost allocation methods—such as overhead and support costs—may affect TDABC’s comparability across institutions, suggesting that standardized approaches could enhance its utility in high-cost, complex procedures like lumbar fusion [22]. Kaplan and Porter (2011) underscore that by implementing a standardized cost framework, health care systems can better identify waste, improve care delivery, and ultimately strengthen the sustainability of VBHC [21]. Adopting these considerations could optimize TDABC’s role in surgical settings and contribute to developing a robust, sustainable framework for VBHC.

## Conclusion

This study demonstrates that surgeon experience and case volume significantly influence costs and outcomes in lumbar fusion surgeries. TDABC proves to be a valuable tool in quantifying these effects and aligning surgical practices with the broader goals of value-based healthcare. As healthcare systems continue to evolve and shift towards models that prioritize cost-effectiveness and outcomes, integrating TDABC into clinical practice will be critical in promoting sustainable and high-quality neurosurgical care.

## Data Availability

No datasets were generated or analysed during the current study.
